# A Novel Touch-Screen, Dual-Language Intervention Program for Older Adults

**DOI:** 10.1093/geroni/igaa057.1839

**Published:** 2020-12-16

**Authors:** W Quin Yow, Hui-Ching Chen, Tharshini Lokanathan, Attila Achenbach, Lucienne Blessing

**Affiliations:** Singapore University of Technology & Design, Singapore, Singapore

## Abstract

Although cognitive training in healthy older adults (OA) has been controversial, specific and isolated cognitive skills such as semantic memory can be improved with appropriate designs. Semantic memory has been considered as a clinical marker for cognitive decline in dementia. The current study, as part of a larger touch-screen dual-language intervention program with cognitive training tools, aims to slow down the rate of cognitive decline in OA with dementia (OwD). A set of neuropsychological tests was conducted before and after the training program. After 24 training sessions over 8-12 weeks, OwD (11 females, 1 male, mean=85.8yo) improved significantly in their verbal working memory (Rey Auditory Verbal Learning Test; RAVLT) while performance of the cognitive-healthy OA (5 females, 3 males, mean=76.3yo) remained the same post-intervention. Our findings suggest that touch-screen technology can help OwD improve their semantic memory. The strengths and limitations of our game design and intervention will be discussed. Part of a symposium sponsored by Technology and Aging Interest Group.

